# Supplementary material for: Polyketide synthase gene clusters in the ericoid mycorrhizal fungus Oidiodendron maius: genome-wide prediction, expression profiling, and functional characterization of knockout mutants
Source: Front Plant Sci. 2026 Sep 16;17:1924430. doi: 10.3389/fpls.2026.1924430 (PMC13623903; doi:10.3389/fpls.2026.1924430)
Supplement: Supplementary file 1 [file SupplementaryFile1.zip › Supplementary Materials/Supplementary Folder 1.zip!/Oidma1_AssemblyScaffolds_Repeatmasked/knownclusterblast/cluster3/jgi.p_Oidma1_21852_mibig_hits.html]

| MIBiG Protein | Description | MIBiG Cluster | MiBiG Product | % ID | % Coverage | BLAST Score | E-value |
| --- | --- | --- | --- | --- | --- | --- | --- |
| AAG27132.1 | Fum6p | BGC0000062 | Fumonisin\_biosynthetic\_gene\_cluster | 35 | 101.454545455 | 612 | 8.9e-175 |
| ACB12553.1 | Fum6 | BGC0000063 | Fumonisin\_biosynthetic\_gene\_cluster | 34 | 100.363636364 | 597 | 2.3e-170 |
| AAM54108.1 | cytochrome\_P450 | BGC0000020 | Ansamitocin\_biosynthetic\_gene\_cluster | 36 | 95.3636363636 | 586 | 9e-167 |
| Solyc07g062520.2 |  | BGC0001319 | alpha-Tomatine\_biosynthetic\_gene\_cluster. | 29 | 37.6363636364 | 128 | 6.7e-29 |
| ABM21573.1 | CrpE | BGC0000975 | Cryptophycin\_biosynthetic\_gene\_cluster | 24 | 39.2727272727 | 114 | 5.8e-25 |
| ACB12560.1 | Fum15 | BGC0000063 | Fumonisin\_biosynthetic\_gene\_cluster | 27 | 32.8181818182 | 98 | 5.7e-20 |
| AGO65988.1 | putative\_cytochrome\_p450 | BGC0000992 | Fusaridione\_A\_biosynthetic\_gene\_cluster | 28 | 21.9090909091 | 92 | 2.4e-18 |
| EER93097.1 | hypothetical\_protein | BGC0000798 | Dhurrin\_biosynthetic\_gene\_cluster | 28 | 20.4545454545 | 79 | 2.7e-14 |
